# 2-Methacryloyloxyethyl phosphorylcholine (MPC)-polymer suppresses an increase of oral bacteria: a single-blind, crossover clinical trial

**DOI:** 10.1007/s00784-018-2490-2

**Published:** 2018-05-16

**Authors:** Natsumi Fujiwara, Hiromichi Yumoto, Koji Miyamoto, Katsuhiko Hirota, Hiromi Nakae, Saya Tanaka, Keiji Murakami, Yasusei Kudo, Kazumi Ozaki, Yoichiro Miyake

**Affiliations:** 1 Department of Oral Microbiology, Tokushima University Graduate School of Biomedical Sciences, 3-18-15 Kuramoto-cho, Tokushima, Tokushima 770-8504 Japan; 2 Department of Oral Healthcare Promotion, Tokushima University Graduate School of Biomedical Sciences, 3-18-15 Kuramoto-cho, Tokushima, Tokushima 770-8504 Japan; 3 Department of Periodontology and Endodontology, Tokushima University Graduate School of Biomedical Sciences, 3-18-15 Kuramoto-cho, Tokushima, Tokushima 770-8504 Japan; 4 Life Science Products Division, NOF Corporation, 4-20-3 Ebisu, Shibuya-ku, Tokyo, 150-6019 Japan; 5 Present Address: Department of Medical Hygiene, Dental Hygiene Course, Kochi Gakuen College, 292-26 Asahi tenjin-cho, Kochi, Kochi 780-0955 Japan; 6 Department of Hygiene and Oral Health Science, Tokushima University Graduate School of Biomedical Sciences, 3-18-15 Kuramoto-cho, Tokushima, Tokushima 770-8504 Japan; 7 Present Address: Department of Oral Health Sciences, Faculty of Health and Welfare, Tokushima Bunri University, 180 Nishihama, Yamashiro-cho, Tokushima, Tokushima 770-8514 Japan; 8 School of Oral Health and Welfare, Tokushima University Faculty of Dentistry, 3-18-15 Kuramoto-cho, Tokushima, Tokushima 770-8504 Japan; 9 Department of Oral Molecular Pathology, Tokushima University Graduate School of Biomedical Sciences, 3-18-15 Kuramoto-cho, Tokushima, Tokushima 770-8504 Japan

**Keywords:** 2-Methacryloyloxyethyl phosphorylcholine, Clinical study, Oral infection, Oral bacteria, *Fusobacterium nucleatum*

## Abstract

**Objectives:**

The biocompatible 2-methacryloyloxyethyl phosphorylcholine (MPC)-polymers, which mimic a biomembrane, reduce protein adsorption and bacterial adhesion and inhibit cell attachment. The aim of this study is to clarify whether MPC-polymer can suppress the bacterial adherence in oral cavity by a crossover design. We also investigated the number of *Fusobacterium nucleatum*, which is the key bacterium forming dental plaque, in clinical samples.

**Materials and methods:**

This study was a randomized, placebo-controlled, single-blind, crossover study, with two treatment periods separated by a 2-week washout period. We conducted clinical trial with 20 healthy subjects to evaluate the effect of 5% MPC-polymer mouthwash after 5 h on oral microflora. PBS was used as a control. The bacterial number in the gargling sample before and after intervention was counted by an electronic bacterial counter and a culture method. DNA amounts of total bacteria and *F. nucleatum* were examined by q-PCR.

**Results:**

The numbers of total bacteria and oral streptcocci after 5 h of 5% MPC-polymer treatment significantly decreased, compared to the control group. Moreover, the DNA amounts of total bacteria and *F. nucleatum* significantly decreased by 5% MPC-polymer mouthwash.

**Conclusions:**

We suggest that MPC-polymer coating in the oral cavity may suppress the oral bacterial adherence.

**Clinical relevance:**

MPC-polymer can be a potent compound for the control of oral microflora to prevent oral infection.

## Introduction

Dental caries, periodontal disease, and oral mucosal lesions are major public health problems worldwide, which are caused by oral biofilm. Systemic diseases, such as cardiovascular disease and complications during pregnancy, have been reported to relate with oral microflora [1, 2]. In particular, aspiration pneumonia has been caused by oral bacteria for elderly people and immunocompromised patients [3, 4]. Oral health is closely related with general health and quality of life (QOL) [5]. These facts indicate that it is important to suppress dental plaque formation and development to maintain QOL.

In the process of dental plaque formation, acquired enamel pellicle forms on hard tissue such as tooth surface [6], and then, early colonizers were primarily composed of Gram-positive species such as *Streptococcus* adhere to the surfaces. Secondary colonizers attach primary bacteria already anchored to teeth or tissues, which is important for the development of dental plaque [7, 8]. Fusobacteria play a central role in physical bridges to mediate the co-aggregation of bacterial cells and promote the anaerobic microenvironment [9]. *Fusobacterium nucleatum* is the predominant species in the healthy oral cavity and markedly increases in the oral cavity with periodontal disease. The inhibition of biofilm formation by periodontopathic bacteria including *F. nucleatum*, therefore, is regarded as an effective strategy for preventing periodontal diseases.

It has been shown that the biological properties of a coating of biocompatible 2-methacryloyloxyethyl phosphorylcholine (MPC)-polymers, which have a phospholipid polar group that mimics a biomembrane, are completely harmless to humans, reducing protein adsorption and bacterial adhesion and inhibiting cell attachment [10–12]. We previously reported that a coating of non-aqueous MPC-polymer on coverslips decreased bacterial adhesion, suppressed biofilm formation, and attributed these effects to the “superhydrophilicity” of MPC-polymer-coated surfaces [13]. MPC-polymer application markedly inhibited both the adherence and biofilm formation of *Streptococcus mutans* on saliva-coated hydroxyapatite and streptococcal adherence to oral epithelial cells and reduced the adherence of *F. nucleatum* to streptococcal biofilms in vitro [14]. In the small-scale clinical trial, mouth rinsing with MPC-polymer inhibited the increase of oral bacterial numbers, especially *S. mutans* in vivo, indicating that MPC-polymer coating in oral cavity can be useful for preventing oral infections including dental caries by preventing microbial adherence to oral surfaces [14]. Clinical effect of MPC-polymer on preventing microbial adherence to oral surfaces needs to verify more details because the previous study was a small-scale trial and parallel study design. The aim of this study is to clarify whether MPC-polymer can suppress bacterial adherence in oral cavity by a crossover clinical trial. We also investigated the number of *F. nucleatum,* which is the key bacterium forming dental plaque, using clinical samples.

## Materials and methods

### Study design

**Fig. 1:**
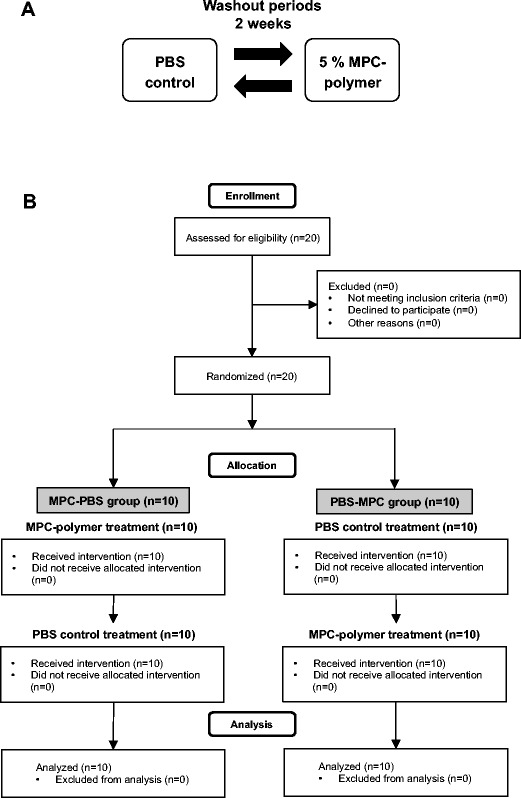
**a** The flow of two treatment and washout period. The study was a randomized, placebo-controlled, single-blind, crossover study, with two treatment periods separated by a 2-week washout period. **b** Flow diagram of this study design phases including enrollment and allocation criteria

The study was a randomized, placebo-controlled, single-blind, crossover study, with two treatment periods separated by a 2-week washout period carried out at Tokushima University between March and July 2016. The flow of two treatments was phosphate buffered saline (PBS, 0.01 M, pH 7.4) as a control followed by 5% MPC-polymer dissolved with sterilized water, or 5% MPC-polymer followed by PBS (Fig. 1a). This clinical trial was approved by the Ethics Committee of Tokushima University Hospital (approval no. 2416).

### Subjects

The sample size calculation was performed on the basis of our previous results [14]. Based on a standard deviation of 0.9 (the fold increase of bacterial number after intervention), a significant level of *α* = 0.05, and a statistical power of 0.9, we calculated that 18 subjects would be necessary to detect a statistically significant and clinically relevant difference of δ = 0.9. The subjects were recruited at Tokushima University. We conducted clinical trial with 20 healthy subjects to evaluate the effect of MPC-polymer mouthwash on oral microflora. Subjects who are wearing orthodontic appliances and removable partial dentures, had any antibiotic therapy in the past 3 months, are pregnant, and are breast-feeding women were excluded. All subjects received the two treatments and were analyzed (Fig. 1b).

### Examination of oral health status

Clinical oral examinations were carried out before this clinical intervention. As a caries experience, we used DMF rate. Periodontal index (PI) [15], oral hygiene index (OHI) [16], and tongue coating status evaluation were also used. In the tongue coating status evaluation, the total area was recorded with a score from 0 to 4, where a score of 0 represented no tongue coating; 1, a thin coating of less than one third of the back of the tongue; 2, a thin coating of less than two thirds of the tongue or less than one-third covered with a thick coating; 3, a thin coating of more than two thirds of the tongue, or less than two thirds covered with a thick coating; and 4, more than two thirds of the tongue covered with a thick coating [17]. Evaluation of all examination indexes were performed by two calibrated and trained examiners (NF and HN).

### Clinical intervention

**Fig. 2:**
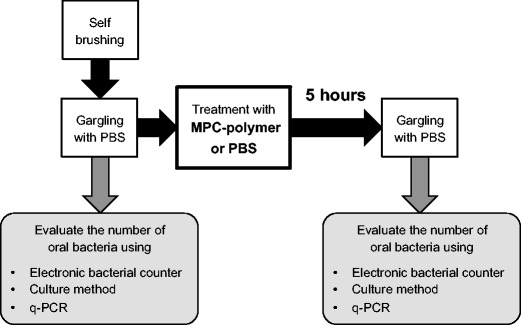
Application regimen of one intervention by using a control or 5% MPC-polymer and items of evaluation using the samples. After the subjects brushed their teeth as usual, we harvested an oral bacterial sample by having them gargle with 5 ml of phosphate buffered saline (PBS) for 20 s. Subjects then are treated with 5 ml of 5% MPC-polymer or PBS (as a control) for 20 s. After 5 h of intervention, an oral bacterial sample was taken again. The subjects were prohibited from eating during the 5-h period. Collected gargling samples were enumerated for the number of bacteria using electronic bacterial counter, a culture method and quantitative real-time polymerase chain reaction (q-PCR)

After the subjects brushed their teeth as usual, we harvested an oral bacterial sample by having them gargle with 5 mL of PBS for 20 s in a similar method that was previously reported [18]. Subjects then were treated with 5 mL of 5% MPC-polymer or PBS (control) for 20 s. At 5 h after treatment with 5% MPC-polymer, an oral bacterial sample was taken again by having them gargle with 5 mL of PBS for 20 s. The subjects were prohibited from eating during the 5-h period. Outcome measures were the number and DNA amounts of oral bacteria before and after intervention by using electronic bacterial counter, culture method, and quantitative real-time polymerase chain reaction (q-PCR) (Fig. 2).

### Counting the number of oral bacteria

The number of total bacteria in the gargling sample before and after intervention was counted by rapid oral bacteria counting system (Panasonic Healthcare Co. Ltd., Osaka, Japan) applying the dielectrophoresis and impedance measurement theory [19]. Fifty microliters of the sample was added in the cup containing its exclusive solution, and the number of total bacteria was automatically counted. The cell numbers in the gargling sample were determined before and after intervention. As the lowest detection limit of this machine is 1 × 10^5^ cells/ml, actual bacterial counts less than this limit were displayed as 1 × 10^5^ cells/ml [20].

The number of bacteria was also counted by a culture method using a spiral plater (model D; Spiral Systems Inc., Cincinnati, OH, USA), which is a device that accurately distributes a liquid sample onto a rotating agar plate for precise bacterial enumeration. Collected samples were diluted 10-fold with saline and 50 μl of diluted samples was inoculated onto agar plates automatically. A blood agar plate (Kyokuto, Tokyo, Japan) supplemented with 5% sheep blood, mitis-salivarius agar plate (Becton Dickinson, Sparks, MD, USA), and mitis-salivarius agar containing 15% sucrose and 3.3 mg/l bacitracin as described previously [21] for total anaerobic bacteria, oral streptococci, and *S. mutans,* respectively, were used. The plates were incubated at 37 °C for 2 days anaerobically using the Anaeropack system (Mitsubishi Gas Chemical, Tokyo, Japan), and the resulting colonies, i.e. CFU, were counted. The numbers of CFU were determined before and after intervention.

### Microbial enumeration by q-PCR

The collected gargled samples were used for q-PCR with the specific primer pairs based on 16S ribosomal RNA. Samples were first centrifuged for 10 min, and their bacterial genomic DNA was extracted and purified using Maxwell® Rapid Sample Concentrator (RSC) DNA FFPE Kit-PKK Custom (Promega Corporation, Wisconsin, USA) and Maxwell® RSC Instrument (Promega Corporation) according to the manufacturer’s instructions. In this study, we did not exclude DNA from dead cells and extracellular DNA before DNA isolation procedure. Real-time PCR was performed with the Fast SYBR® Green Master Mix (Applied Biosystems, Foster City, CA, USA) using the StepOnePlus Real Time PCR System (Applied Biosystems). The following specific primers were used to amplify fragments of *Universal*: 5′-TCCTACGGGAGGCAGCAGT-3′ (sense) and 5′-GGACTACCAGGGTATCTAATCCTGTT-3′ (antisense) [22], and 16S rRNA of *F. nucleatum*: 5′-CGCAGAAGGTGAAAGTCCTGTAT-3′ (sense) and 5′- TGGTCCTCACTGATTCACACAGA-3′ (antisense) [23]. A purified PCR product amplified from *F. nucleatum* was used for generating a quantitative standard curve. Amplification conditions of the q-PCR were 95 °C for 10 min for initial denaturation, 40 denaturation cycles at 95 °C for 3 s and annealing/extension at 60 °C for 30 s. For the relative quantification, the copy numbers of both bacterial genes were normalized to the copy number of the 16S rRNA gene using threshold cycle (ΔΔCt) method. Threshold cycle values and data analyses were performed by StepOne™ Software v2.2 (Applied Biosystems). The bacterial copy numbers of 5 h after treatment with 5% MPC-polymer were compared to that of 5 h after treatment with PBS (control).

### Statistical analysis

All statistical analyses were evaluated by Student’s *t* test which is a common parametric test for comparing two groups with normal distributions. q-PCR was conducted in triplicate and statistical analyses were performed. All analyses were performed using SPSS, version 24.0 (SPSS Japan Inc., Tokyo, Japan). Differences were considered significant when probability values were less than 5%.

## Results

### Oral health status of subjects

We investigated the clinical oral status using DMF rate, PI, OHI, and tongue coating status against 20 subjects before the intervention (Table 1). The mean age of 20 subjects was 30 ± 9.25, and sex distribution of the subjects was 8 males and 12 females. Average rate of DMF index in the subjects was 21.4 ± 15.7%. Averages of PI and OHI were 0.45 ± 0.6 and 0.98 ± 1.1, respectively. Average of tongue coating status was 1.53 ± 0.6.

**Table 1:** Baseline characteristics and clinical oral health status of the subjects

	Total subjects (*n* = 20)	MPC-PBS group (*n* = 10)	PBS-MPC group (*n* = 10)
Age (years)	30.0 (± 9.3)	30.9 (± 10.1)	29.1 (± 8.8)
Female/male	12/8	5/5	7/3
DMF rate (%)	21.4 (± 15.7)	16.5 (± 18.0)	25.8 (± 12.9)
Periodontal index (PI)	0.45 (± 0.6)	0.5 (± 0.6)	0.2 (± 0.3)
Oral hygiene index (OHI)	0.98 (± 1.1)	1.1 (± 1.0)	0.5 (± 1.0)
Tongue coating test	1.53 (± 0.6)	1.6 (± 0.5)	1.4 (± 0.7)

Average (standard deviation)

### Effects of mouthwash with 5% MPC-polymer on the number of oral bacteria

**Fig. 3:**
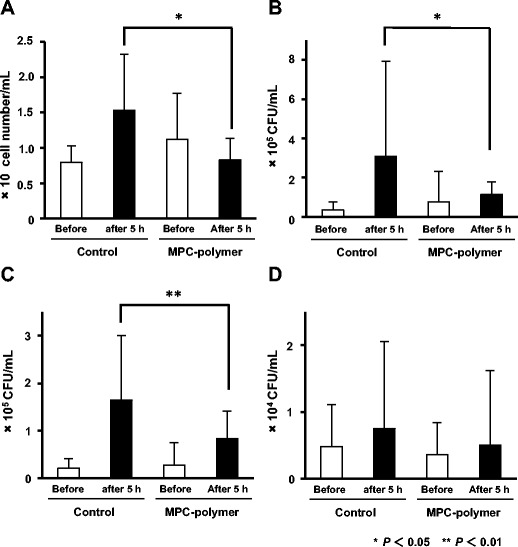
The effect of mouthwash with 5% MPC-polymer coating on oral bacterial numbers. The bacterial numbers in the gargled sample were counted by an electronic bacterial counter and a culture method. In the culture method, gargling samples were spread on agar plates supplemented with 5% sheep blood, mitis-salivarius (MS) agar plate and MS agar containing 15% sucrose and 3.3 mg/l bacitracin (MSB) plate, using a spiral plater device. Colony-forming units (CFUs) were counted after anaerobic incubation at 37 °C for 2 days. The data using **a** an electronic bacterial counter and **b** agar plates supplemented with 5% sheep blood, **c** MS agar plates and **d** MSB plates in culture procedure were shown. The numbers of cells and CFU in control and 5% MPC-polymer-treated group before intervention showed no significant difference. Data represent the mean ± S.D. *P < 0.05 and **P < 0.01

We investigated the effect of mouthwash with MPC-polymer on bacterial colonization in the oral cavity of the subjects. The total bacterial numbers after 5 h in MPC-polymer-treated group were significantly lower than that of control group (*P* < 0.05) by automatic counting using a rapid oral bacteria quantification system (Fig. 3a). Data obtained by culture method also showed that the numbers of CFU after 5 h of total bacteria and oral streptococci in MPC-polymer-treated group were significantly lower than that of control group (*P* < 0.05 and *P* < 0.01, respectively) (Fig. 3b, c). In addition, The CFU counts of *S. mutans* in MPC-polymer-treated group were also lower than that of control group, but there was no statistical significance (Fig. 3d). We also confirmed that the numbers of cells and CFU in control and 5% MPC-polymer-treated group before intervention showed no significant difference.

**Fig. 4:**
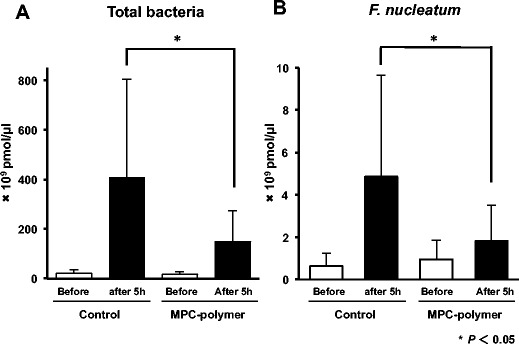
The effect of treatment with 5% MPC-polymer on the copy numbers of 16S rRNA gene of **a** total bacterial and **b** *Fusobacterium nucleatum* numbers by q-PCR. Samples were enumerated for the number of total bacteria and *F. nucleatum* by q-PCR using specific primer. Before intervention, the copy numbers of 16S rRNA gene of total bacteria and *F. nucleatum* show no significant difference. Data represent the mean ± S.D. of three independent experiments. *P < 0.05

The copy numbers of the 16S rRNA gene of total bacteria and *F. nucleatum* were evaluated by q-PCR. The copy numbers of total bacteria and *F. nucleatum* in control and 5% MPC-polymer-treated group showed no significant difference before intervention. The copy numbers of total bacteria and *F. nucleatum* after 5 h in 5% MPC-polymer-treated group were significantly lower than those in control group (*P* < 0.05) (Fig. 4).

## Discussion

It is well known that microorganisms often survive within biofilms, which results in environmental problems and various infectious diseases [24]. During the biofilm formation and its maturation, co-aggregation and co-adhesion of bacteria are critical [9]. Inhibition of bacterial adherence, therefore, has been considered an effective strategy for prevention of infectious diseases. To destroy the biofilm in the oral cavity, many studies have been conducted on the effective strategies in dental hygiene practice. As a mechanical method, tooth brushing and tongue scraping are used to remove microorganisms. Mouthrinses and toothpastes containing antibacterial compounds, such as chlorhexidine, cetylpyridinium chloride (CPC), and triclosan, are commonly used to prevent the growth and the biofilm formation of bacteria. Currently used disinfectants, however, induce adverse effects, such as extrinsic brown staining of teeth and restorations, toxic to mucous membranes, burning sensation, and mouth irritation [25]. Taking into account these disadvantages of the disinfectants, a novel strategy to inhibit the bacterial adherence and dental plaque formation is required.

Here, we demonstrated the inhibitory effects of mouthwash with 5% MPC-polymer coating in the oral cavity on the number of oral bacteria by a randomized, crossover clinical study. Importantly, MPC-polymer has been approved by the Food and Drug Administration (FDA) and applied for various purposes, such as contact lenses and cosmetics. We previously have shown that (i) MPC-polymer coating to plastic coverslips reduces retention of human pathogenic microorganisms including *Staphylococcus aureus*, *S. mutans*, *Pseudomonas aeruginosa*, and *Candida albicans* in vitro [13], and (ii) MPC-polymer application markedly inhibited both the adherence and biofilm formation of *S. mutans* on saliva-coated hydroxyapatite and oral epithelial cells [14]. These findings show that superhydrophilicity of MPC-polymer-coated surfaces, but not disinfectant action, may inhibit the adherence of pathogenic bacteria to teeth and/or oral mucosa. In addition, MPC-polymer coating has a role for reducing the protein adsorption. However, the salivary proteins included in the acquired enamel pellicle, such as histatin and statherin, have protective effects of demineralization, prevention of acidic dissolution of the teeth, and antibacterial/antifungal effects [26–28]. In the future, therefore, the development of MPC-polymer with the ability of absorbance of selective salivary proteins are desired.

Fusobacteria play a central role as physical bridges to mediate the co-aggregation of bacterial cells and promote an anaerobic microenvironment. *F. nucleatum* is a predominant and key bacterium in the dental plaque formation and closely associates with other periodontal pathogens [9]. Production of hydrogen sulfide by *F. nucleatum* is known to be associated with halitosis [29]. MPC-polymer coating inhibits the adherence of *F. nucleatum* to streptococcal biofilms in vitro [14]. Here, we showed that 5% MPC-polymer gargle significantly suppressed the increase of *F. nucleatum* to 50% (Fig. 4b). These findings suggest that mouthwash with MPC-polymer coating may suppress the adherence of *F. nucleatum* to the oral cavity and may inhibit the maturation of dental plaque. *F. nucleatum* has been reported to be involved in the development of colon cancer via activation of oncogenic signaling, recruitment of tumor-infiltrating immune cells, and interference of the host immunity [30–32]. The control of *F. nucleatum* is important to reduce the risk of colon cancer as well as oral infectious diseases.

To count the bacterial number in the oral cavity, we employed the gargling method. Although it is almost impossible to count the actual bacterial number in the oral cavity, we can estimate the actual number from a sample that is appropriately taken. It has been reported that the bacterial number obtained by the gargling method well reflects the number of bacteria that inhabit in the oral cavity [18]. The bacteria adhered to oral mucosa, tooth surfaces, and tongue, therefore, can be collected by the gargling and the bacterial number in the gargling sample may be well correlated to the whole number of bacteria inhabiting in the oral cavity. To assess the number of oral bacteria in gargling sample, we used a device adopted for rapid oral bacteria quantification system (electronic bacterial counter). Although the electronic bacterial counter has a limit of the detection of bacterial counts (less than 10^5^ cells/ml), the number of microorganisms in the saliva usually exceeds 10^5^ cells/ml [20]. The result by an electronic counting method did not show a highly significant difference in comparison with the result by a culture method (Fig. 3a, b). A difference in bacterial viability may be one of the reasons for which there was low significant difference. Moreover, an electronic bacterial counter is lower sensitivity than the culture method. However, the data evaluated by electronic bacterial counter was well correlated with the data evaluated by a culture method (*r*^2^ = 0.7814). As the electronic bacterial counter is simple and easy-to-use operation, this device is useful for counting oral bacteria. Therefore, we can easily check the number of oral bacteria in gargling sample from elderly people at home and compromised hosts in a hospital. We will examine the effect of MPC-polymer coating for elderly people and compromised hosts in the future.

In conclusion, we suggest that MPC-polymer coating in the oral cavity may suppress the oral bacterial adhesion. We believe that MPC-polymer is a potent compound for the control of oral microflora to prevent oral infection including dental caries, periodontal diseases, and halitosis. In this study, we focused on the oral bacterial adherence after MPC-polymer treatment. To know the initial microorganism recovery after the intervention, we will evaluate the clinical oral health status including the status of tongue coating.

As MPC-polymer treatment protected human oral keratinocytes from the damage induced by CPC [33], MPC-polymer can be used for the purpose on the protection from the damage of oral mucosa in combination with other commercially available disinfectants.
